# Quasi-two-dimensional superconductivity in FeSe_0.3_Te_0.7_ thin films and electric-field modulation of superconducting transition

**DOI:** 10.1038/srep14133

**Published:** 2015-09-18

**Authors:** Zhu Lin, Chenguang Mei, Linlin Wei, Zhangao Sun, Shilong Wu, Haoliang Huang, Shu Zhang, Chang Liu, Yang Feng, Huanfang Tian, Huaixin Yang, Jianqi Li, Yayu Wang, Guangming Zhang, Yalin Lu, Yonggang Zhao

**Affiliations:** 1 Department of Physics and State Key Laboratory of Low-Dimensional Quantum Physics, Tsinghua University, Beijing 100084, China; 2 Beijing National Laboratory for Condensed Matter Physics, Chinese Academy of Sciences, Beijing 100190, China; 3 Collaborative Innovation Center of Quantum Matter, Beijing 100084, China; 4 CAS Key Laboratory of Materials for Energy Conversion, Hefei National Laboratory for Physical Sciences at the Microscale & National Synchrotron Radiation Laboratory, University of Science and Technology of China, Hefei 230026, China

## Abstract

We report the structural and superconducting properties of FeSe_0.3_Te_0.7_ (FST) thin films with different thicknesses grown on ferroelectric Pb(Mg_1/3_Nb_2/3_)_0.7_Ti_0.3_O_3_ substrates. It was shown that the FST films undergo biaxial tensile strains which are fully relaxed for films with thicknesses above 200 nm. Electrical transport measurements reveal that the ultrathin films exhibit an insulating behavior and superconductivity appears for thicker films with T_c_ saturated above 200 nm. The current-voltage curves around the superconducting transition follow the Berezinskii-Kosterlitz-Thouless (BKT) transition behavior and the resistance-temperature curves can be described by the Halperin–Nelson relation, revealing quasi-two-dimensional phase fluctuation in FST thin films. The Ginzburg number decreases with increasing film thickness indicating the decrease of the strength of thermal fluctuations. Upon applying electric field to the heterostructure, T_c_ of FST thin film increases due to the reduction of the tensile strain in FST. This work sheds light on the superconductivity, strain effect as well as electric-field modulation of superconductivity in FST films.

Heterostructures composed of superconductors and ferroelectrics (SC/FE) are important for studying the coupling between superconductivity and ferroelectricity, especially the modulation of superconductivity by ferroelectricity, as well as applications of devices^1^. It has been shown that superconductivity can be modulated by ferroelectric field effect^2^,^3^ or biaxial strain related to the converse piezoelectric effect of FE^4^. For biaxial-strain-effect study, substrates with different mismatches with the film have been widely used^5^,^6^,^7^,^8^. In this case, besides the biaxial strain effect, other effects may be induced due to the different interfaces and mismatch-related defects, etc.^4^. While for the biaxial strain related to the converse piezoelectric effect of FE in the FE-related heterostructure, it applies to the same sample with continuous and reversible (sometimes non-volatile) nature^9^,^10^, and overcomes the disadvantages of the biaxial strains induced by substrate-film lattice mismatch. Therefore, it is a very unique and advantageous approach to study the biaxial strain effect on superconductivity by means of SC/FE heterostructures. Pb(Mg_1/3_Nb_2/3_)_0.7_Ti_0.3_O_3_ (PMN-PT) exhibits ultra-high piezoelectric behavior^11^, so it has been widely used in the biaxial-strain-effect study of FE-based heterostructures, especially ferromagnetic/PMN-PT heterostructures for electric-field control of magnetism^9^,^12^. Iron-based superconductors have attracted much attention recently. However, the study of biaxial strain effect in heterostructures composed of iron-based superconductors and FE is rather limited. Trommler *et al.* reported the biaxial strain effect of BaFe_1.8_Co_0.2_As_2_/PMN-PT (ref. 4) with a small modification of the superconducting transition temperature (T_c_). FeSe_1-x_Te_x_ (FST) system is very unique among the iron-based superconductors since it has a simple crystallographic structure with stacking of FeSe_4_ tetrahedra layers and arsenic-free^13^,^14^,^15^. More interestingly, it has been shown that a pressure of 8.9 GPa enhanced the T_c_ of FeSe up to 36.7 K and T_c_ even increased up to 65 K in a single-layered FeSe film grown on SrTiO_3_^16^,^17^,^18^. It should be mentioned that the superconducting tetragonal phase of Fe_x_Se only forms in a temperature range of 300 °C–440 °C and composition window x = 1.01–1.025. This extreme sensitivity to synthesis conditions makes the growth of FeSe films difficult by PLD. FeSe_1-x_Te_x_, when Se substituted by Te, however, forms the same tetragonal structure and is easily grown by PLD. Since its T_c_ is higher and it is more stable than FeSe, there have been a lot of work on FeSe_1-x_Te_x_ films^19^. Therefore, it is interesting to study the biaxial strain effect of FST in FST/PMN-PT heterostructures. Up to now, there has been no report on FST/PMN-PT heterostructures although there have been some work on FST thin film grown on non-ferroelectric substrates^5^,^20^,^21^. Moreover, there is a large lattice mismatch between FST (*a* = 3.814 Å) and PMN-PT (*a* = 4.02 Å)^22^,^23^, so tensile strain is induced in FST and this strain decreases from the interface to the surface of FST. Since tensile strain decreases T_c_ of FST^5^, the T_c_ of FST is expected to increase from the interface to the surface of FST. As a result, the ultrathin region near the surface of FST has the highest T_c_, which provides a route to study the two-dimensional (2D) superconductivity. It should be mentioned that so far the dimensionality of superconductivity in iron-based superconductors is still an open question^24^,^25^.

In this paper, we report the structural and superconducting properties of FeSe_0.3_Te_0.7_ thin films with different thicknesses grown on PMN-PT substrates. It was shown that the FST films undergo biaxial tensile strains and the strain relaxes with the increase of film thickness and is fully relaxed for films with thicknesses above 200 nm. Electrical transport measurements reveal that the ultrathin films exhibit an insulating behavior and superconductivity appears for thicker films with T_c_ saturated above 200 nm. The current-voltage curves around the superconducting transition follow the Berezinskii-Kosterlitz-Thouless (BKT) behavior, while the resistance-temperature curves can be described by the Halperin–Nelson formula, revealing the quasi-two-dimensional phase fluctuation in FST thin films. The Ginzburg number decreases with increasing film thickness indicating the decrease of the strength of thermal fluctuations for thicker films. Electric field increases T_c_ of FST thin film, which is attributed to the reduction of the tensile strain in FST film.

## Results

**Figure 1:**
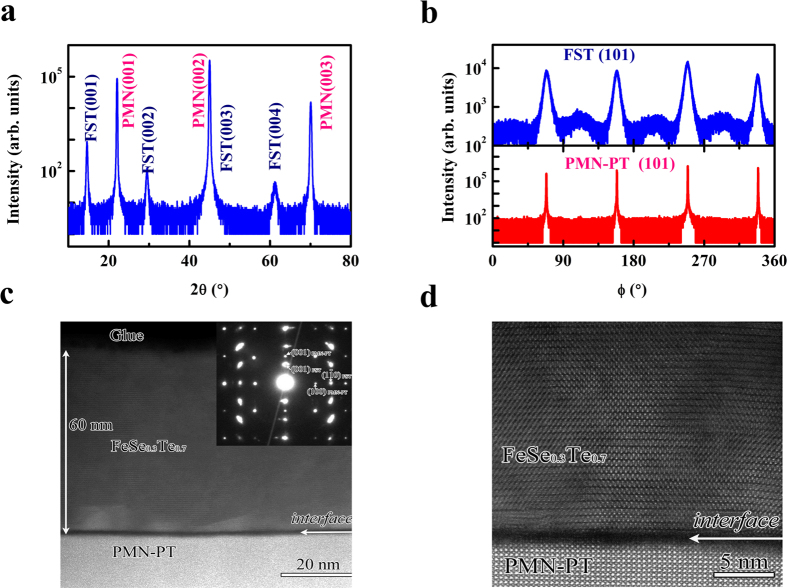
X-ray diffraction patterns and Transmission electron microscopy (TEM). (**a**) X-ray diffraction patterns of θ−2θ scans for FST thin film grown on a (001)-cut PMN-PT substrate. (**b**) ϕ-scan of FST (101) peak and PMN-PT (101) peak. (**c**) The low-magnification STEM image of FST/PMN-PT. The inset shows the SAED pattern of FST/PMN-PT. (**d**) High-magnification STEM cross-sectional image of FST/PMN-PT.

Figure 1(a) is the X-ray diffraction patterns (XRD) of θ−2θ scans for FST thin film with a thickness of 200 nm. It can be seen that FST film shows *c*-axis orientation with (00l) peaks. The results of ϕ scan of FST (101) peak and PMN-PT (101) peak are shown in Fig. 1(b). Similar to that of PMN-PT, there are also four peaks separated by 90° for FST. This fourfold symmetry shows *ab* plane alignment indicating epitaxial growth of the FST films. However, there is a blunt hump peak between 101 peak and it indicates the existence of some 45° in-plane rotation in FST film, suggesting that the in-plane FST structure consists of grains with high-angle-tilt grain boundaries. This may be related to the large lattice mismatch and poor bonding between FST and PMN-PT. It should be mentioned that this 45° in-plane rotation often happened in the films of iron-based superconductors grown by PLD when lattice mismatch between films and substrates is large. For example, there is a 45° in-plane rotation in FeSe_0.5_Te_0.5_ film grown on MgO (001)^26^ and in Ba(Fe, Co)_2_As_2_ film grown on bare LSAT(001)^27^. Figure 1(c) shows the low magnification STEM image and the corresponding selected-area diffraction (SAED) pattern from the interface area for a 60 nm thick FST film. Two sets of electron-diffraction spots, arising from the FST film and the substrate, respectively, can be unambiguously indexed based on the PMN-PT structure (a cubic cell with the lattice parameter of 4.02 Å)^23^ and FST structure (a tetragonal cell with the lattice constant of *a* = 3.814 Å and *c* = 6.157 Å)^22^. This pattern clearly exhibits the orientation relationship of [001]_FST_//[001]_PMN-PT_. Figure 1(d) shows a high resolution STEM image illustrating the cross-sectional structure of a FST/PMN-PT sample. It is clear that the thickness of the amorphous layer between the film and substrate is less than 1 nm; however defect structure can be often observed in the areas close to the interface (Fig. S1 of Supplementary information). The presence of this defect structure can be attributed to the large lattice mismatch between FST and PMN-PT.

**Figure 2:**
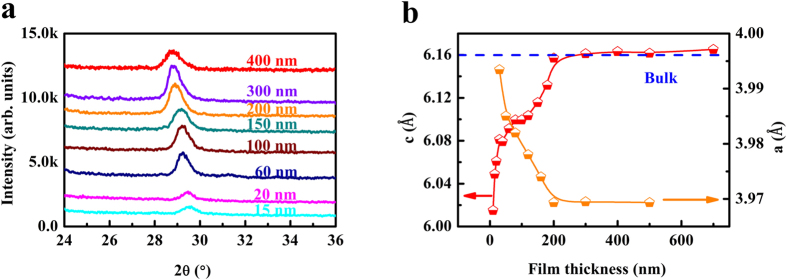
X-ray diffraction patterns and lattice parameters of the FST films with different thicknesses. (**a**) The (002) diffraction peak of the FST films with different thicknesses. (**b**) Variation of the lattice parameters of *a* and *c* with film thickness.

Variation of the (002) peak for the XRD patterns of FST films with different thicknesses is shown in Fig. 2(a), which reveals that the peak shifts to low angles. From Fig. 2(a), we can get the dependence of lattice parameter *c* on film thickness according to the Bragg equation and the result is presented in Fig. 2(b). It shows that lattice parameter *c* increases as the film thickness increases, and reaches the bulk value for films with thicknesses above 200 nm^22^. This behavior can be understood by considering the relaxation of the biaxial tensile strain in FST originated from the lattice mismatch between FST (*a* = 3.814 Å) and PMN-PT (*a* = 4.02 Å)^22^,^23^. The in-plane lattice parameter, measured by grazing incidence X-ray diffraction (GIXRD), is also shown in Fig. 2(b). The XRD patterns can be found in the Supplementary information (Fig. S2). As expected, it shows the opposite behavior compared with the lattice parameter *c*.

**Figure 3:**
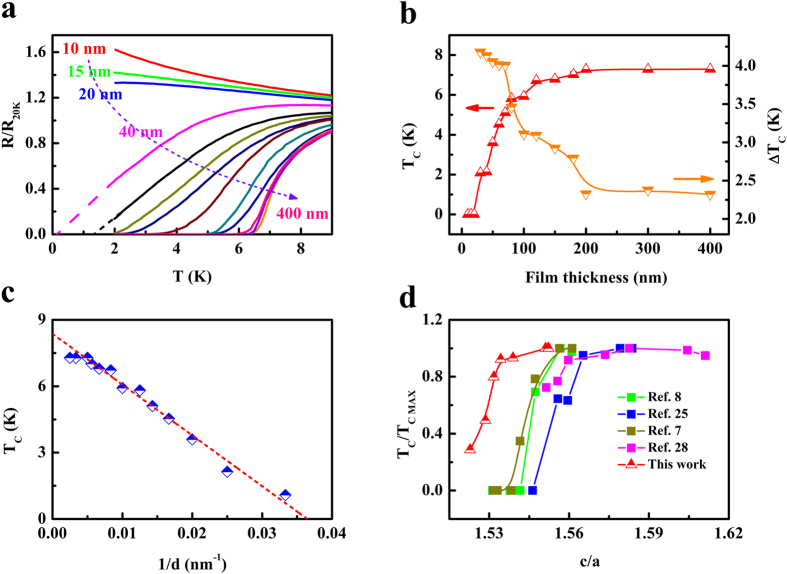
Electrical transport properties of FST films with different thicknesses. (**a**) R-T curves at low temperatures for FST films with different thicknesses (normalized to *R*_*20K*_). (**b**) Variation of T_c_ and ΔT_c_ with film thickness. (**c**) Plot of T_c_ vs. *1/d*. (**d**) Variation of T_c_ for FST films with *c*/*a*.

Since FST films with different thicknesses undergo different biaxial tensile strains, it is interesting to explore their transport and superconducting properties. Figure 3(a) shows the temperature dependence of resistance (R-T curve) at low temperatures for FST films with different thicknesses. At small thicknesses, the R-T curves exhibit an insulating behavior, which may be related to the defects located near the FST/PMN-PT interface (Fig. S1). The R-T curves can be described by the weak localization model (see details in Fig. S3 of Supplementary information). For films with large thicknesses, superconductivity appears and T_c_ increases with film thickness. Figure 3(b) is the variation of T_c_ (middle point of the superconducting transition) and the transition width (the temperature difference for resistance drops between 10% and 90%) ΔT_c_ with film thickness. It can be seen that T_c_ increases and ΔT_c_ decreases respectively with film thickness and becomes saturated for films thicker than 200 nm. The plot of T_c_ vs. *1/d* (*d* is the thickness of FST film) is shown in Fig. 3(c). For films with small thicknesses, the dependence of T_c_ on *1/d* can be roughly described by a linear relation. Similar behavior has been found in YBa_2_Cu_3_O_7-x_ thin films grown on SrTiO_3_ (ref. 28). By extrapolating the linear fit to T_c_ = 0 K, the corresponding thickness was obtained to be about 30 nm, which can be regarded as the “dead layer” for superconductivity. On the other hand, one can also get the “dead layer” from the dependence of conductance on film thickness at temperatures above T_c_ (Fig. S4). This “dead layer” is likely related to the defect structure (Fig. S1) and the interfacial effect. Figure 3(d) is the variation of T_c_ with the ratio of lattice parameters *c* and *a* (*c*/*a*). It can be seen that T_c_ changes only within a certain range of *c*/*a*, and remains unchanged outside this range. In Fig. 3(d), we also plotted the data of FST films grown on different substrates or with different film thicknesses reported in the literature^7^,^8^,^29^,^30^ and found that they follows the same trend with a small shift. Further work is needed to understand the relation between T_c_ and *c*/*a*.

**Figure 4:**
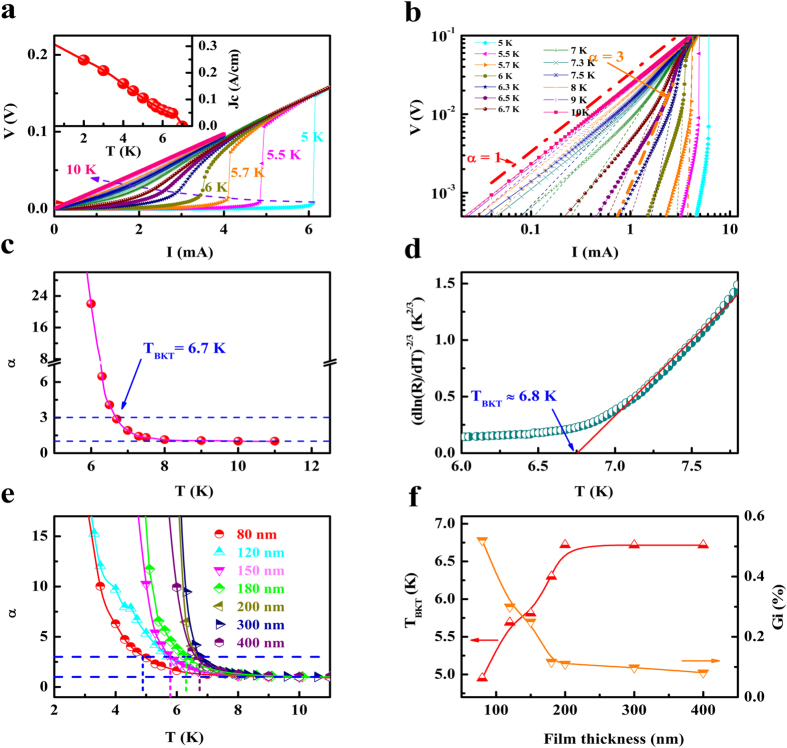
Berezinskii-Kosterlitz-Thouless (BKT) transition behavior of FST films. (**a**) *V-I* curves for the 200 nm thick FST film measured at different temperatures. The inset shows the temperature-dependent critical current density. (**b**) Plot of *V-I* data in a log–log scale. The short dash lines are power-law fits of the data in the BKT transitions at different temperatures. The red line corresponds to a 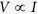 behaviour while the orange long line corresponds to a 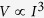 behavior. (**c**) Temperature dependence of the power-law exponent *α*. (**d**) *R*-*T* curve with a [dln(*R*)/d*T*]^−2/3^ versus *T* plot. The red line is the behaviour expected for a BKT transition with T_BKT_ ≈ 6.8 K. (**e**) Temperature dependence of the power-law exponent *α* for FST with different thicknesses. (**f**) Variation of *T*_*BKT*_ and *Gi* with FST film thickness.

Up to now, the dimensionality of superconductivity in iron-based superconductors is still an open question^24^,^25^. For 2D superconductivity, electrical transport properties show the signature of BKT transition occurring at a characteristic temperature (*T*_BKT_), below which vortices and antivortices are bound in pairs^31^. At the BKT transition, the voltage-current (*V-I*) follows the power-law dependence as 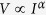 with α = 3 at *T*_BKT_ (ref. 28). Figure 4(a) is the *V-I* curves for the 200 nm thick FST film measured at different temperatures around the T_c_ and the inset is the temperature dependence of critical current density obtained from it. In order to check whether voltage-current follows the power law, the *V-I* data are plotted in the log-log scale as shown in Fig. 4(b). The straight lines in this plot show the power-law behavior and the slope equals to α. The value of α equals to 1 at high temperatures, indicating an ohmic characteristic, and increases with decreasing temperature and reaches 3 at T_BKT_, corresponding to the BKT transition. Figure 4(c) is the temperature dependence of the power-law exponent α, obtained from the fits in Fig. 4(b). It can be seen that the value of α reaches 3 at T = 6.7 K, which is the *T*_BKT_ of FST film, and increases rapidly below *T*_BKT_. Similar treatments were carried out for FST films with other thicknesses (Fig. S5 of Supplementary information) and they also show 2D superconducting behavior. The temperature dependences of the power-law exponent α for FST films with different thicknesses are shown in Fig. 4(e) and the corresponding values of *T*_BKT_ are shown in Fig. 4(f). On the other hand, for temperatures above *T*_BKT_, the resistance is expected to follow 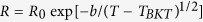 (ref. 31), where *R*_0_ and *b* are material-dependent parameters. As shown in Fig. 4(d), the temperature dependence of resistance is consistent with this expectation and gives *T*_BKT_ ≈ 6.8 K, comparable to that obtained from the *V-I* data. Similar treatments were carried out for FST films with other thicknesses (see details in Fig. S6 of Supplementary information). Therefore, the above analyses strongly suggest 2D superconductivity in FST films. Moreover, paraconductivity analysis also suggests 2D superconductivity in FST films (S7 of Supplementary information). For superconductors, the strength of thermal fluctuation can be characterized by the Ginzburg number 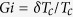
25,32, which is the relative temperature width of a superconducting fluctuation region, and T_c_ is the mean-field temperature (*T*_BKT_ < T_c_ < *T*_MF_) shifted by the superconducting fluctuations^24^. The values of *Gi* for FST films with different thicknesses are shown in Fig. 4(f). The details for getting *Gi* for FST films with different thicknesses can be found in S7 of Supplementary information. It can be seen that the value of *Gi* decreases with the increase of film thickness and becomes saturated for films thicker than 200 nm, suggesting that the thermal fluctuation decreases for thicker films. We also studied the 2D superconductivity in FST films grown on CaF_2_ since the value of T_c_ for FST films grown on CaF_2_ can reach 15 K (ref. 5) and they also show 2D superconductivity (S6 of Supplementary information).

**Figure 5:**
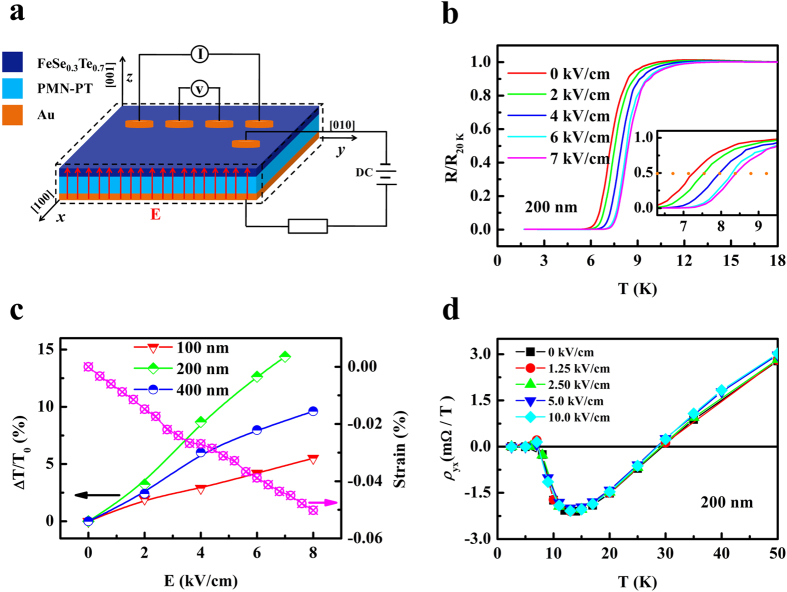
Electric-field modulation of superconductivity of FST film. (**a**) Schematic of the sample and the experimental configuration. (**b**) Superconducting transition curves for a 200 nm thick FST film under different electric fields. The inset shows the magnification around the transition. (**c**) Variation of T_c_ for FST films with different thicknesses and strain of PMN-PT with electric field. (**d**). Temperature dependence of the Hall resistance of FST film under different electric fields.

Since FST thin films were grown on ferroelectric PMN-PT substrates, it is interesting to explore electric-field modulation of superconductivity. Figure 5(a) is the schematic of the sample and the experimental configuration. Figure 5(b) is the superconducting transition curves for a 200 nm thick FST film under different electric fields. We also measured superconducting transition curves for 100 nm and 400 nm thick FST films under different electric fields (S9 of Supplementary information). The superconducting transition shifts to higher temperatures with increasing electric field and the variation of T_c_ with electric field for FST films with different thicknesses are shown in Fig. 5(c). It can be seen that the 200 nm thick film shows the largest change. In order to understand the origin of this electric-field modulation of superconductivity, we also measured the electric-field-induced strains and the results are also shown in Fig. 5(c). It can be seen that T_c_ increases with decreasing strain, indicating that the change of T_c_ is related to the variation of strain. Moreover, we carried out electric-field-induced lattice strain in the PMN-PT substrate and FST film by measurements of XRD under electric fields and obvious changes were observed (S8 of Supplementary information). It should be mentioned that the electric-field modulation of superconductivity can not be attributed to the electric-field effect because the Hall effect measurements did not show any change of carrier density as shown in Fig. 5(d). Moreover, electric-field effect should be minor considering the large thickness and metallic nature of FST thin films since the Deybe screening length is about 1-2 unit cells for metal^33^. Therefore, the electric-field modulation of superconductivity for FST film can be attributed to the electric-field-induced strain, which transfers to FST film, leading to reduction of the lattice-mismatch-induced tensile strain in FST film. This results in the increase of T_c_.

## Discussion

**Figure 6:**
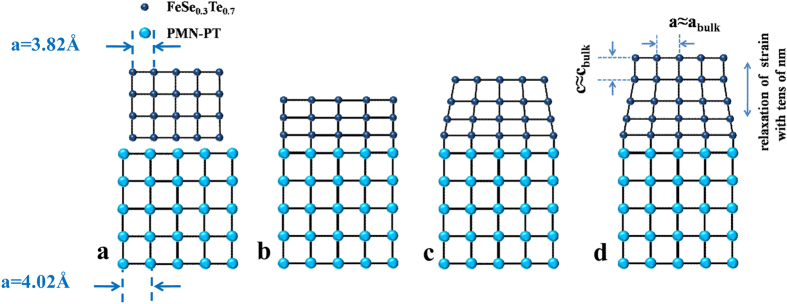
Schematic of strain relaxation in FST of FST/PMN-PT heterostructure. (**a**) Lattice of FST and PMN-PT. (**b**) Ultrathin FST films. (**c**) FST films with intermediate thicknesses. (**d**) Thicker FST films.

There are two ways to realize 2D superconductivity^34^. In the case when the interplane coupling in the structure of layered superconductor becomes very weak, the superconductor behaves essentially as independent 2D superconducting planes. The other case is when the perpendicular correlation length of the superconductor is larger than its thickness. Figure 6 is the schematic of strain-relaxation model^35^,^36^,^37^, which can account for the behaviors of FST thins films with different thicknesses. Since there is a large lattice mismatch between FST (*a* = 3.814 Å) and PMN-PT (*a* = 4.02 Å)^22^,^23^, FST films are subjected to tensile strains. For the ultrathin FST films, roughly speaking, they are fully strained as shown in Fig. 6(b) and the samples exhibit an insulating behavior. This insulating behavior originates from the degradation of films due to the defect structure and the interfacial effect. For FST films with intermediate thicknesses (Fig. 6(c)), the tensile strain decreases from the region close to the interface to the surface of the film. Since tensile strain decreases the superconducting transition temperature (T_c_) of FST^5^, the value of T_c_ is expected to increase from the interface to the film surface. As a result, the top region near the surface of FST has the highest T_c_, resulting in 2D superconductivity. With further increase of thickness for FST film (Fig. 6(d)), the tensile strain is fully relaxed for the top layer of FST films and the value of T_c_ becomes saturated. It should be mentioned that when we measure the resistance of FST films with thicknesses below 200 nm, the current will mainly flow in the region near the film surface. So, 2D superconductivity and corresponding T_BKT_ mainly reflect the nature of this region. However, for FST films with thicknesses above 200 nm, the current will mainly flow in the top thick layer of the strain relaxed region and the 2D superconductivity and corresponding T_BKT_ mainly reflect the nature of this region. The emergence of 2D superconductivity in the thicker FST films suggests its intrinsic nature for FST films. It should be mentioned that 2D superconductivity has also been reported in single crystals and thick films of high T_c_ superconductors, such as 500 nm thick FeSe films^25^, single crystals of cuprates^38^,^39^ and 500 nm thick cuprate films^40^. The 2D superconductivity in these systems can be understood by considering their layered structures and the weak interplane coupling since the coherence lengths perpendicular to the planes are very short^25^,^38^,^39^,^40^. As shown in Fig. 4(f), the value of *Gi* decreases with the increase of film thickness and becomes saturated for films thicker than 200 nm. This can be understood by considering that only the top thin layer for the FST films with intermediate thicknesses becomes superconducting at the transition temperature. So their thermal fluctuations should be more remarkable compared with the thicker FST films. For the electric-field modulation of superconducting transition of FST thin films, it can be attributed to the reduction of the tensile strain via the transfer of piezostrain in PMN-PT to FST.

In summary, FST films grown on PMN-PT are subjected to biaxial-tensile strain, which fully relaxes for films with thicknesses above 200 nm. Electrical transport measurements reveal that the ultrathin films exhibit an insulating behavior and superconductivity appears for thicker films with T_c_ saturated above 200 nm. The current-voltage curves around the superconducting transition follow the BKT transition behavior and the resistance-temperature curves can be described by the Halperin–Nelson relation, revealing 2D superconductivity in FST thin films. The Ginzburg number decreases with increasing film thickness indicating the decrease of the strength of thermal fluctuations. Upon applying electric field to the heterostructure, T_c_ of FST thin film increases due to the reduction of the lattice-mismatch-induced tensile strain. This work is helpful for understanding the superconducting behaviors of FST and manipulation of superconductivity via electric fields.

## Methods

FeSe_0.3_Te_0.7_ films were grown on one-side-polished (001)-oriented Pb(Mg_1/3_Nb_2/3_)_0.7_Ti_0.3_O_3_ (PMN-PT) substrates under vacuum (10^−4^ Pa) by pulsed-laser deposition (PLD) using a KrF laser (wavelength 248 nm). The target for PLD was prepared by solid state reaction method. Powder materials of Fe (3N purity), Se (3N purity), and Te (5N purity) with a nominal composition of FeSe_0.3_Te_0.7_ were fully mixed. The well-mixed powders were cold pressed into discs, and then sealed in an evacuated quartz tube with a pressure less than 10^−4^ torr and heat treated at 300 °C for 5 h, then 600 °C for 12 h. The product was then mixed and cold pressed again, then heated at 650 °C for 24 h. During film deposition, the substrate temperature was set at 275 °C. The frequency of the laser beam was 3 Hz and the pulse energy density on the target was about 3 J/cm^2^. After deposition, the films were cooled down to room temperature under vacuum. The thicknesses of the films were measured by a Dektak 6M stylus profiler. The quality of the FeSe_0.3_Te_0.7_ films was characterized by four-circle X-ray diffraction (XRD) on a Rigaku D/max-RB X-ray diffractometer with a Cu K_α_ radiation. To measure the in-plane lattice parameters, grazing incidence X-ray diffraction (GIXRD) was performed on a four-circle diffractometer with a Ge (220) × 2 incident-beam monochromatorand 0.5° in plane receiving parallel slit (RigakuSmartLab Film Version with an in-plane arm for GIXRD, Cu-*Kα* radiation). The grazing angles were set to *α*_*i*_ = *α*_*f*_ = 0.25°, which corresponds to the critical angle of the film-air interface measured by XRR. Samples for cross-section TEM were prepared using a standard procedure consisting of gluing, cutting, mechanical polishing, dimpling, and ion milling. STEM observations were performed in the JEOL ARM200F equipped with double aberration correctors and operated at 200 kV. Electrical transport property of the films was measured by means of a superconducting quantum interference device (MPMS 7 T, Quantum Design) with four-probe method. For strain measurements, the strain gauge was pasted on PMN-PT with glue M-Bond 610, and kept at 120 °C for 2 h to strengthen the paste effect. The Hall resistance measurement under different electric fields was carried out using standard four-probe ac lock-in method, with the current flowing in the film plane and applied magnetic field perpendicular to the plane. To avoid chemical contamination to the sample, the Hall bar geometry is scratched by hand. For contact, small pieces of indium (In) is pressed onto the top surface of the sample mechanically.

## Additional Information

**How to cite this article**: Lin, Z. *et al.* Quasi-two-dimensional superconductivity in FeSe_0.3_Te_0.7_ thin films and electric-field modulation of superconducting transition. *Sci. Rep.* **5**, 14133; doi: 10.1038/srep14133 (2015).

## Supplementary Material

Supplementary Information
