# Supplementary material for: Quasi-two-dimensional superconductivity in FeSe0.3Te0.7 thin films and electric-field modulation of superconducting transition
Source: Sci Rep. 2015 Sep 18;5:14133. doi: 10.1038/srep14133 (PMC4585655; doi:10.1038/srep14133)
Supplement: Supplementary Information [file srep14133-s1.doc]

Supplementary information

Quasi-two-dimensional superconductivity in FeSe_0.3_Te_0.7_ thin films and electric-field modulation of superconducting transition

Zhu Lin,^1,3^ Chenguang Mei,^1,3^ Linlin Wei,^2,3^ Zhangao Sun,^2,3^ Shilong Wu,^2,3^ Haoliang Huang,^4^ Shu Zhang,^1,3^ Chang Liu^1,3^, Yang Feng^1,3^, Huanfang Tian,^2,3^ Huaixin Yang,^2,3^, Jianqi Li,^2,3^ Yayu Wang,^1,3^, Guangming Zhang^1,3^, Yalin Lu^4^ & Yonggang Zhao^1,3^*

*^1^Department of Physics and State Key Laboratory of Low-Dimensional Quantum Physics, Tsinghua University, Beijing 100084, China*

*^2^Beijing National Laboratory for Condensed Matter Physics, Chinese Academy of Sciences, Beijing 100190, China*

*^3^Collaborative Innovation Center of Quantum Matter, Beijing 100084, China*

*^4^CAS Key Laboratory of Materials for Energy Conversion, Hefei National Laboratory for Physical Sciences at the Microscale & National Synchrotron Radiation Laboratory, University of Science and Technology of China, Hefei 230026, China*

Correspondence and requests for materials should be addressed to Y. Z. (ygzhao@tsinghua.edu.cn)

**S1. Defect structure in the areas close to interface for FST thin films**

In order to understand the effect of the structure on the superconducting property of the FST films. We also did high resolution STEM measurment and the result is shown in Fig. S1. It can be seen that defect structures exist near the interface. These defect structures, probably related to the large lattic mismatch between PMN-PT and FST, may account for the insulating behavior of FST films with small thicknesses and the existence of the “dead layer”.

**Figure S1** | High-resolution STEM image showing the details of the interface of epitaxial growth of FST film. The local defect structures are indicated by arrows.

**S2. In-plane lattice parameters of FST films grown on PMN-PT with different thicknesses.**

We have done the grazing incident X-ray diffraction (GIXRD) to measure the in-plane lattice parameters, which can directly obtain the interspace of FST (100)/(010) at serval tens nanometer of surface and avoid intense signal from the substrate, the results are shown in Fig. S2.

**Figure S2** | (a) The (200) diffraction peak of the FST films with different thicknesses. (b) Variation of the lattice parameters of *a* with film thickness.

**S3. Weak localization for electronic transport in FST films with small thicknesses**

As shown in Fig. 3(a), FST films with small thicknesses show insulating behavior, which is likely related to the defect structures near the interface via localization effect. In order to understand the electronic transport behavior of the FST films with small thicknesses, we fitted the R-T curve of 10 nm thick FST film by using different models. Figure S3(a) is the temperature dependence of resistance for 10 nm thick FST film grown on PMN-PT. It turns out that Mott’s variable range hopping for the two dimensional case with $R=R_{0}\exp(T_{0}/T)^{1/3}$(Ref. 1) gave the best fit at low temperatures with $R_{0}\approx170\text{ }\Omega$, and $T_{0}\approx8.43\text{ }K$as shown in Fig. S3(b).

**Figure S3** | Mott’s variable-range-hopping (M-VRH) fitting of the R-T curve. (a) Temperature dependence of resistance for 10 nm thick FST film. (b) M-VRH (2D) fitting at low temperatures for the R-T curve of FST film.

**S4. “Dead layer” thickness for FST films on PMN-PT deduced from the conductance**

The thickness of the “dead layer” can also be deduced from the dependence of conductance on film thickness (t) as demonstrated for manganite thin films^2^. As shown in Fig. 3(a), the resistance of FST film decreases with increasing thickness with a insulator-metal (superconductor) transition. Figure S4 is the variation of conductance at 15 K with film thickness for FST. The conductance shows a linear dependence on thickness. By extrapolating to zero conductance, the thickness of the “dead layer” is deduced to be about 20 nm, which is roughly consistent with the results of T_c_ measurement (Fig. 3(c)) and HRTEM (Fig. S1). Therefore, thicker films are preferred for good superconductiving properties.

**Figure S4 |** Variation of conductance with film thickness at 15 K.

**S5. BKT transition in FST films with different thicknesses**

Figure S5 is the plot of the voltage-current data in a log–log scale for FST films with different thicknesses and Fig. S6 is the *R*-*T* curves with a [dln(*R*)/d*T*]^-2/3^ versus *T* plot for FST films with different thicknesses. From the fitting results, we can get the values of T_BKT_ of the 80 nm, 120 nm, 150 nm, 180 nm, 200 nm, 300 nm, 400 nm to be 5.1 K, 5.8 K, 5.9 K, 6.25 K, 6.7 K, 6.6 K, 6. 8 K, respectively.

**Figure S5 |** Voltage-current data plotted in a log–log scale for FST films with different thicknesses. The red short dash lines are power-law ﬁts of the data in the BKT transitions at different temperatures. The orange line corresponds to a $V\propto I$ behaviour and the black line to a $V\propto I^{3}$ behavior.

**Figure S6 |** *R*-*T* curves with a [dln(*R*)/d*T*]^-2/3^ versus *T* plot for FST film with different thicknesses. The red solid line is the behavior expected for a BKT transition with T_BKT_.

**S6. BKT transition in FST films grown on CaF_2_(001)**

From previous report^6^, we know that CaF_2_(001) is more suitable than PMN-PT(001) to grow FST film with better superconductivity. In order to analyze 2D superconductivity properties clearly, we have grown two FST films on substrate CaF_2_(001) with thicknesses of 100 nm and 200 nm respectively, and the results are shown in Fig. S7 and Fig. S8. The results reflect that T_c_ of FST films grown on CaF_2_(001) is higher than that grown on PMN-PT(001). 2D superconductivity hehaviour exist in both films with T_BKT_ =10.85 K for 100 nm thick film and T_BKT_ =13.83 K for 200 nm thick film, respectivily.

**Figure S7 |** R-T curve and 2D superconductivity of FST film grown on CaF_2_(001) with a thickness of 100 nm.

**Figure S8 |** R-T curve and 2D superconductivity of FST film grown on CaF_2_(001) with a thickness of 200 nm.

**S7. 2D superconductivity revealed by paraconductivity and Ginzburg numbers for FST films with different thicknesses**

With Friedmann’s improved method^3^ for two-dimensional Aslamazov-Larkin (2D AL) theoretical model and three-dimensional Aslamazov-Larkin (3D AL) theoretical model^4^, the paraconductivity can be described by the following formulas.

(2D)

(3D)

**Figure S9** | Fitting of temperature dependence of paraconductivity with different theoretical models for a 200 nm thick film. (a) 2D AL model. (b) 3D AL model.

Where *σ’* means paraconductivity, *d* is the relevant layer thickness and *ε* = ln(*T/T*_MF_) (*T*_MF_ is the mean-field transition temperature), we got the best fitting result by choosing different data ranges for both models. Figure S9 shows fitting of temperature dependence of paraconductivity with different theoretical models for a 200 nm thick film. It can be seen that both 2D and 3D models can fit the data well. However, the fitting of 3D model gives a coherence length of 0.0457 nm. Such a small coherence length is in contradiction to the 3D nature and is unrealistic. While the fitting of 2D model results in *d* = 1.047 nm, which is reasonable. Therefore, the analysis of paraconductivity data suggests 2D superconducting nature of FST film.

Then we can get the values of Gi number of for FST films with different thicknesses by solving equations of *T*_BKT_ *= T*_c_*(1-4*Gi*) and (*T*_MF_ *– T*_c_)/*T*_c_ *=* -2*Gi**ln*Gi* given by Larkin et al^5^, where *T*_MF_ was obtained from above fitting of temperature dependence of paraconductivity with 2D AL model and *T*_BKT_ was got from BKT theory (see S5). For FST film with a thickness of 200 nm, *T*_MF_ = 6.86 K and *T*_BKT_ = 6.72 K, then we have *Gi* = 1.1x10^-3^. The values of *Gi* for FST films with different thicknesses are shown in Fig. 4(f).

**S8. Electric-ﬁeld-induced out-of-plane strains in the PMN-PT substrate and FST film as a function of electric ﬁeld.**

In order to understand the effects of the electric-field-induced lattice strain on the superconductivity properties of the FST film, we have examined the electric-field-induced lattice strain in the PMN-PT substrate and FST film by measurements of XRD under electric fields. Figure R8a shows that the PMN-PT(003) reflection shifts to lower $2\theta$ angles with increasing electric field from 0 to 9 kV/cm, which implies that the lattice parameter *c* of the PMN-PT substrate has been elongated by electric fields. Figure S10b shows that FST(004) reflection also shifts to lower $2\theta$ angles with increasing electric field from 0 to 9 kV/cm, suggesting that electric-field-induced lattice strain in the PMN-PT substrate has transferred to FST film successfully. The induced out-of-plane strain $\varepsilon_{zz}$ can be estimated using $\varepsilon_{zz}=\frac{c(E)-c(0)}{c(0)}$ where $c(E)$ and $c(0)$ are the lattice parameter *c* obtained from XRD measurements with and without an electric field *E*, respectively. We plot $\varepsilon_{zz}$ of PMN-PT and FST film as a function of electric field as shown in Fig. 10c. From the Poisson effect, we can deduce that the in-plane lattice will contract under an electric field *E*. So compared with the strain state of the FST film with *E*=0 kV/cm, the PMN-PT substrate imposes an in-plane compressive strain on the FST film when a positive electric field is applied to the positively polarized PMN-PT substrate.

**Figure S10** | XRD patterns for the PMN-PT(003) reflection (a) and FST(004) reflection (b) under electric ﬁelds. (c) Electric-ﬁeld-induced out-of-plane strains in the PMN-PT substrate and FST film as a function of electric ﬁeld.

**S9. Electric-field modulation of superconductivity of FST film with thickness 100 nm and 400 nm.**

In order to understand the superconductivity properties of FST film under different strain states more deeply, we measured the strain effect of T_c_ in films with the thicknesses of 100 nm and 400 nm and the results are shown in Fig. S11. Figures S11a and S11c are the superconducting transition curves for a 100 nm and 400 nm thick FST films under different electric fields, respectively. The superconducting transition shifts to higher temperatures with increasing electric field and the variation of T_c_ with electric field are shown in Fig. S11(b) and Fig. S11(d). The relationship of different thickness FST film between T_c_ and electric field is similar, but slope is different.

**Figure S11** | Superconducting transition curves for the 100 nm thick FST film (a) and 400 nm thick FST film (c) under different electric fields. The inset shows the magnification around the transition; Variation of T_c_ for 100 nm thick FST film (b) and 400 nm thick FST film (d) with electric field.

**References**

1. Mott, N. F. Conduction in Glasses Containing Transition Metal Ions. *J. Non-Cryst. Solids* **1**, 1-17 (1968)
2. Sun, J. Z. *et al.* Thickness-dependent magnetotransport in ultrathin manganite films. *Appl. Phys. Lett.* **74**, 3017 (1999).
3. Friedmann, T. A. *et al.* In-plane paraconductivity in a single crystal of superconducting YBa_2_Cu_3_O_7-x_. *Phys. Rev. B* **39**, 4258 (1989).
4. Aslamazov, L. G. & Larkin, A. I. The influence of fluctuation pairing of electrons On the conductivity of normal metal. *Phys. Lett.* **26A**, 238 (1968).
5. Larkin, A. & Varlamov, A. Theory of Fluctuations in Superconductors (New York: Oxford University Press) 2005.
6. Hanawa, M. *et al.* Empirical Selection Rule of Substrate Materials for Iron Chalcogenide Superconducting Thin Films. *Jpn. J. Appl. Phys.* **51**, 010104 (2012).
